# Pregabalin Misuse and Abuse in Jordan: a Qualitative Study of User Experiences

**DOI:** 10.1007/s11469-017-9813-4

**Published:** 2017-09-15

**Authors:** Amneh Al-Husseini, Mayyada Wazaify, Marie Claire Van Hout

**Affiliations:** 1 Department of Biopharmaceutics and Clinical Pharmacy, School of Pharmacy, The University of Jordan (UJ), Amman, Jordan; 2 Public Health Institute, Liverpool John Moore’s University, Liverpool, UK

**Keywords:** Abuse, Community pharmacy, Jordan, Pregabalin

## Abstract

Pregabalin is currently approved for the treatment of epilepsy, generalized anxiety disorder, neuropathic pain, and fibromyalgia. A qualitative study was undertaken in Jordan, where concerns have been raised about its unprescribed availability in community pharmacies and thereby its abuse. Semi-structured interviews were conducted with all patients with a history of pregabalin use in two Jordanian addiction treatment centers. All were male patients aged 21–30 years (*n* = 11). The majority was poly-drug abusers and had a previous history of substance abuse (tramadol, Captagon, synthetic cannabinoids, and marijuana)*.* Six key themes emerged from a content textual analysis which centered on *pregabalin and other drugs*; *the pregabalin effect*; *poly-pharming and pregabalin intoxication*; *trajectories*, *patterns*, *and routes of administration*; *dependence and withdrawal*; and *sourcing of pregabalin*. The study underscores the need for continued pharmacovigilance to manage and address suspected abuse, along with community pharmacist and patient education regarding abuse liability and related harms.

Abuse of prescription and over-the-counter (OTC) narcotics is a global issue (Hughes et al. 1999; Casati et al. 2012). OTC medications are those that can be procured from pharmacies or other retail outlets without a prescription while prescription medications are those that cannot be dispensed unless a prescription written by a physician is available (Lessenger and Feinberg 2008). Although the sale of OTC medications from community pharmacies may help individuals to self-treat minor ailments, save time, and minimize the effort of patients and physicians, some of these medicines can be abused, which subsequently may lead to addiction (Cooper 2013). The risks of addiction to prescription and OTC medications rise when they are used in means other than prescribed (e.g., at higher doses, by other routes of administration, or mixed with alcohol or other drugs). Adverse health and social consequences related to the misuse of prescription and OTC narcotics, particularly analgesics, stimulants, and CNS depressants, have been steadily worsening worldwide (National Institute on Drug Abuse (NIDA) 2014).

The most commonly reported prescription narcotics to be abused worldwide are stimulants such as methylphenidate and central nervous system (CNS) depressants such as sedatives (benzodiazepines) or some anticonvulsants like clonazepam (National Institute on Drug Abuse 2014) or pregabalin (Loftus and Wright 2014). Of interest for this study are pregabalin, which is an analog of the gamma-aminobutyric acid (GABA) mammalian neurotransmitter, and its structurally related compound gabapentin known as α2,δ ligands. They act as inhibitory modulators of neuronal excitability that reduce ectopic neuronal activation of hyperexcited neurons while normal activation remains unaffected (Papazisis and Tzachanis 2014). Pregabalin is approved for the treatment of partial epilepsy, generalized anxiety disorder, peripheral and central neuropathic pain, and fibromyalgia with an accepted dosage range of 150 to 600 mg/day (Papazisis and Tzachanis 2014). Pregabalin was classified as schedule V of the Controlled Substances Act (CSA) in 2005 in the USA (Drug Enforcement Administration, Department of Justice 2005).

In Jordan, where the study was undertaken, like other countries in the Middle Eastern region, one can buy any medicine without a prescription with the exception of controlled drugs (Wazaify et al. 2016). This open access linked with relatively low-priced products and abundance of community pharmacies is speculated to contribute to greater levels of misuse, abuse, and substance use disorders, which are all related to medicine (Albsoul-Younes et al. 2010). Concerns for medicine abuse have been frequently reported in community pharmacies in Jordan since 2006. Products of concern have included benzodiazepine sedative hypnotics, followed by anticholinergics and anti-Parkinson’s drugs (Albsoul-Younes et al. 2010).

Recent pharmacovigilant trends reported by the Jordan Food and Drugs Administration (JFDA) show the diversion and abuse of the antiepileptic pregabalin and certain ophthalmic drops with sympathetic, antihistamine, or anticholinergic properties (e.g., cyclopentolate) not previously mentioned in Jordan in 2006 (Wazaify et al. 2016). In 2014, a formal statement about the restriction of pregabalin products dispensed in community pharmacies in Jordan was released, whereby pregabalin-containing products were added to a list of restricted drugs requiring a medical prescription for dispensing. This list included drugs with abuse liability but not scheduled as controlled drugs, such as opioids, opoid derivatives, or opioid-containing preparations (Jordan Food and Drugs Administration 2014). Alprazolam as an example was rescheduled in 2013 from *prescription-only medicine* to the controlled schedule III status (Albsoul-Younes et al. 2010). In 2017, another regulatory announcement by the JFDA was released to include pregabalin preparations in the restricted drug list (Jordan Food and Drugs Administration 2017). This is the first study in Jordan which aimed to explore and describe the experiences of those in addiction treatment with a history of pregabalin use and abuse.

## Methods

A qualitative study underpinned by phenomenology was designed to explore and describe contextualized narratives of patient experiences of pregabalin use and abuse and achieve a wider exploration of pregabalin pathways and realities of pregabalin abuse. Ethical approval was granted by the IRB at the Ministry of Health. A qualitative interview instrument was designed by the team who had research and professional expertise in drug addiction, pharmacology, and community pharmacy practice and was based on a systematic review of extant literature on pregabalin misuse and abuse. Questions centered on the participants’ experience with pregabalin, pregabalin effects, patterns and pathways of use and abuse, and experiences of sourcing the drug.

Semi-structured interviews were conducted with patients with a history of pregabalin use in the two governmental Jordanian addiction treatment centers, the National Addicts Rehabilitation Center (run by the Ministry of Health) and the Center for Drug Addiction Treatment (run by the Public Security Directorate). All eligible subjects with pregabalin abuse problems were approached in early 2017 and asked to take part in the study. Several participants were excluded due to withdrawal severity or intoxication. An audio recorder was used during the interviews which were conducted at the treatment centers, after obtaining verbal consent from the participants. All interviews were then transcribed verbatim and translated into the English language.

The data was managed using the software program QSR NVivo 11 Starter (Qualitative Solutions and Research 2011). The phenomenological approach used content and thematic analyses (Holsti 1969; Babbie 2010) in order to identify and present areas of similar and contrasting opinions within the pregabalin misuse and abuse experience. The analytical strategy commenced with several reads of the interview transcripts, which was followed by simple and axial coding conducted by the team in consultation to generate macro groupings, emergent themes, and categories. The identified groups, paragraphs, and sentences were broken down into several codes of key incidents, concepts, and relationships. A certain level of synchronic reliability was achieved, whereby two or more perspectives between the narratives were in relative agreement as to the *lifeworld* experience (Husserl 1970) of pregabalin abuse.

## Results

Six key themes emerged from a narrative analysis, which centered on *pregabalin and other drugs*; *the pregabalin effect*; *poly-pharming and pregabalin intoxication*; *trajectories*, *patterns*, *and routes of administration*; *dependence and withdrawal*; and *sourcing of pregabalin*.

### Participant Profile

A total of 11 patients were interviewed. All participants were male, and the most frequent age category was between 21 and 30 years old. More than half of the participants were single. The most frequent level of education among participants was secondary education. Only seven participants were employed (see Table 1).

**Table 1:** Participant profiles

Items	Case numbers
Marital status	
1—Married	8, 9, 10
2—Single	1, 2, 3, 4, 5, 6, 11
3—Divorced	7
Age	
1—≤ 20	3, 11
2—21–30	1, 2, 5, 6, 8, 10
3—31–40	7, 9
4—Over 40	4
Gender	
Male^a^	All male
Drugs of abuse	
Hashish	1, 2, 3, 6, 7, 8, 9, 10
Marijuana	1, 2, 6
Synthetic hashish (Joker)	1, 5, 9, 10, 7
Captagon	3, 4, 6, 7, 9
Sedatives^b^	1, 2, 5, 6, 7, 10
Anti-Parkinson^c^	5, 6, 7
Alcohol	4, 7
Opioid^d^	1, 2, 4
Cocaine	4
LSD	4
Oxethazaine (oxetacaine)	1
Pregabalin	All patients
Duration of drug abuse^e^	
1–2 years	5, 11
2–5 years	1, 2, 3, 10
> 5 years	4, 6, 7, 8, 9
Education level	
1—Primary school	7, 9, 10
2—Secondary school	4, 5, 11
3—University/postgraduate	1, 2, 3, 6, 8
Occupation	
Employed	9, 10, 11
Unemployed	1, 2, 8, 6
Private business	3, 4, 5, 7

^a^The females in the addiction treatment center are treated as an outpatient

^b^Sedatives: e.g., bromazepam, alprazolam, clonazepam, and diazepam

^c^Anti-Parkinson: e.g., benzhexol and procyclidine

^d^Opioid: e.g., oxycodone, tramadol, and heroin

^e^Duration of drug abuse in general

### Pregabalin and Other Drugs

All participants with one exception were poly-drug users. The majority used natural hashish. The second most common substances used were synthetic cannabinoids (known in Jordan as *Joker*), Captagon, and alprazolam. Marijuana, tramadol, and clonazepam were used by three participants, and two participants used benzhexol (trihexyphenidyl), procyclidine, and alcohol.

I began with natural hashish. Afterwards, I started with marijuana and other sorts of medical pills like Xanax®, tramadol, Oxicane®, and Roxy®. The last thing I used was Lyrica®. I tried Joker, but Lyrica® is more difficult to stop than the Joker because I used to take large quantities (Participant 1, 22 years old, male).

Only one participant was taking pregabalin on its own with no previous history of drug abuse and observed,

I used to take pills named Galica® and no other drugs. This material itself will lead you to take other drugs just like weed! If I stayed using this drug, I would have ended up trying other types of drugs. That is why I came here for treatment (Participant 11, 19 years old, male).

The majority of participants were first introduced to pregabalin from a friend or colleague. More than half of them (*n* = 9/11) experimented for the first time with friends when using other drugs.

From my friends, as I was already a Joker [synthetic cannabinoid] and hashish addict, we used to always take drugs as a group and they (his friends) told me this (pregabalin) was better than Joker. They meant because it is a medical substance (Participant 10, 27 years old, male).

Two participants tried it first as an alternative to tramadol or benzohexol.

I knew it (pregabalin) by an Emeriti friend who used to take it, as an alternative to tramadol. Tramadol is a controlled drug, but these are not and any pharmacy would sell them. I usually take big quantities and I came here to the center on Thursday evening after taking one whole packet of Lyrica® 14 pills (Participant 4, 42 years old, male).

I learned about it (pregabalin) through my friend. He advised me to try and I liked it. It gives a good mood like the mood of Baltane® (benzhexol). First, I took two pills and they changed my mood, and the next day I demanded more (Participant 7, 35 years old, male)

For two participants, the first use of pregabalin was in the context of treatment for addiction. One participant used it as he viewed the drug as a legal substitute for another drug, and another used pregabalin as treatment for synthetic cannabinoid withdrawals.

Now, the idea prevailing among the addict community is that pregabalin can treat addiction, so we thought that it would be legal and normal. We knew it was used for disc treatment, and later on, we came to know that some countries use it for the treatment of addiction. But I took it as a substitute for other materials (Participant 6, 22 years old, male).

This happened by accident. Someone told me it (pregabalin) would treat Joker powder addiction, and I took it. It treated the agitation of the powder only for two days and then it became useless on the third day (Participant 9, 35 years old, male).

Several participants commented that the lack of detection of pregabalin in urine screening methods was a motive for use.

In general, we concentrated on Lyrica® as it doesn’t appear in the urine and we were afraid of antinarcotics department personnel. That’s why we liked Lyrica® (Participant 4, 42 years old, male).

Others commented on its legal status and easy route to access.

Better than Joker and other materials as it is a legal drug (Participant 10, 27 years old, male).

### The Pregabalin Effect

The pregabalin effect reported by each participant varied, depending on the product used, the dose, and whether the use occurred with other poly-substances. Descriptions of the initial euphoric pleasurable effect were similar in all narratives.

At first, I felt sluggish, sleepy, fatigue, with leg pain and shaky hands and many other things happened like foggy eyes but mainly I felt sleepy. The second time you take it (Lyrica®: pregabalin) you feel very active, you want to work, just like the action of Captagon. You feel active and have dry mouth, your hands peel, the body feels dehydrated, and you feel you want to drink something sweet or like Redbull (energy drink). Also, I have reached the stage of cheerfulness with the feeling of happiness which is well-being. Now on continuous taking of Lyrica®, one starts to forget and mingles between the subjects when talking to someone (Participant 3, 20 years old, male).

I can’t describe it, you feel relaxed, too lazy, and it (pregabalin) makes you sleepy. When you stay awake you will go on eating and talking profusely, you became too social. When you take three pills 150 mg, you can talk to anybody without interruption probably for 2 hours (Participant 2, 23 years old, male).

The main perceived positive outcomes of pregabalin use centered on its effect in making users sociable and talkative with others.

It made me change the way I treat people; I became very kind and social which is very far from my personality. Mainly, I had the energy to talk non-stop without knowing where all these words came from (Participant 11, 19 years old, male).

The majority appeared to take this one step further in attempting to reach intoxication.

At first it makes you sleepy, and then it elevates your mood and boosts your energy. But when you start to take 5–6 pills at once, you don’t feel sleepy, you feel active and energetic (Participant 5, 22 years old, male).

The vast majority of participants described noticing that smoking cigarettes increased and was more satisfying when using pregabalin.

I smoke one packet a day, but when I take it (pregabalin), I smoke 3–4 packets non-stop (Participant 1, 22 years old, male).

Smoking is more satisfying with Epigab®. I smoke three and a half packets instead of two when I take Epigab® (Participant 5, 22 years old, male).

### Poly-pharming and Pregabalin Intoxication

Some participants described poly-pharming with other substances to enhance the euphoric effect. More than half of the participants combined pregabalin with natural hashish.

When taking Lyrica® with hashish, they boost each other’s effect (Participant 3, 20 years old, male).

Drinks like energy drinks, Coca Cola®, sweet tea, and coffee were commonly mixed with pregabalin to enhance its stimulant outcomes as described by all participants.

I learnt something from an Emirati, that if you drink Coca Cola® which includes carbon dioxide in it along with Lyrica® it increases its effect. Coca Cola® is well-known combination with Lyrica®. If you search the internet you see, for example, whoever takes Lyrica® always drinks a large Coca Cola® with it to reach its effect faster. The reason is that it contains sugar, and sugar always boosts the effect of Lyrica® (Participant 1, 22 years old, male).

Other participants described mixing pregabalin with different drugs like tramadol, Lexotanil®, synthetic cannabinoid (Joker), alprazolam, and Captagon in order to potentiate its effect.

I was taking it (pregabalin) with the Prazin®, Saliba (clonazepam), and Kemadrin®. They made me confused, but at the same time they gave me a good mood. Also, I started to forget events immediately and talk to myself. Everything I took made me dissociated and unaware of the things around me (Participant 7, 35 years old, male).

Some described the combination with alcohol as dissociative.

Drinking alcohol along with pregabalin will lead to a disaster. I tried it and I hit a wall. That is why I came here to the center (Participant 5, 22 years old, male).

With a can of beer and within 5 minutes, the action sets in. I lose consciousness. Sometimes when awake, I find myself in the police detention room, yet I had no idea what I have committed (Participant 7, 35 years old, male).

Many participants described how pregabalin use in poly-drug-taking episodes contributed to cognitive impairment and detachment from worries and concerns.

It (pregabalin) impaires my thinking and makes me feel like I don’t understand what people are saying; it rambles my thoughts (Participant 7, 35 years old, male).

It (pregabalin) dissociates you from reality, you would feel normal, happy without any worries (Participant 1, 22 years old, male).

Experiences of severe dissociation and overdose as a result of the compounding effects of poly-substances used with pregabalin were common.

I came to the center on Thursday. I was taking Lyrica® strip and Joker powder and passed out for four days and woke up yesterday morning. The nurse told me that he tried to talk to me, but I had no reaction or response and since I came here I am not able to sleep (Participant 4, 42 years old, male).

I went to the university and met an addict who persuaded me to take marijuana. Then I went home and on the roof, it happened with me for the first time, I had an overdose and slept on the ground. I didn’t know what happened as I also took Lyrica® with it (Participant 1, 22 years old, male).

### Trajectories, Patterns, and Routes of Administration

Three different user trajectories were identified, with the majority using pregabalin for less than 1 year, some using between 1 and 3 years, and some more than 3 years. More than half of the participants were abusing pregabalin on a daily basis. Three were using pregabalin every 2 to 3 days in order to reduce tolerance.

I take it (pregabalin) every other day because if I took it daily nothing will happen (Participant 1, 22 years old, male).

Repeated dosage within drug-taking episodes was common.

I began with Lyrica® 75 mg; then I started to increase it to 150 and sometimes 300 like Epigab® 300. I used to take 3–4 pills till I went into deep sleep. If I took it in the morning, I usually took 2–3 pills and at night 2 pills, then 150 and thereafter 300 (4–5 pills), and so on. The highest dose I reached was the last time, I took a complete strip of 14 pills (300), not all at once, but on the same day (Participant 2, 23 years old, male).

I started with Lyrica® at a dose of 300 mg. First, I used to take 3–4 pills, then one strip. The highest dose I took was 28 pills of Lyrica®, at one time, I mean two strips (Participant 8, 29 years old, male).

Participants described using personal experience and experimentation with dosage and intervals of use to boost the effect and elongate its duration. Most used it only by swallowing the capsule. Others experimented with insufflation or by pouring the capsule contents directly into the mouth.

I used to take it (pregabalin) with a glass of water and sometimes I tried to open it and put the drug in my mouth right away. I tried to sniff it once, but it had no effect (Participant 1, 22 years old, male).

At first I used to swallow pregabalin pills; then I started to take it by chewing, suckling, and then swallowing it. Later on, I started to open it and pour the powder in my mouth (Participant 11, 19 years old, male).

### Dependence and Withdrawals

The majority of participants were not aware of the adverse health consequences of pregabalin abuse.

Regarding the negative consequences, I only knew it (pregabalin) would harm the kidneys, and then I came to know about other consequences during the treatment. It impacts the nerves, genitalia, brain, kidney, increase/decrease heart beats, and would raise blood pressure. But the addictive effects I knew about them from my friends (Participant 3, 20 years old, male).

Many described awareness of dependence and the all-consuming nature of seeking and using pregabalin.

The worst part is that I lost everything. I no longer have confidence in myself. My whole thinking was devoted to the idea of getting money to buy Lyrica® (Participant 2, 23 years old, male).

I used to make different excuses to get money. For example, I would say I had to pay for university transport fare or fill up my car in addition to other things (Participant 3, 20 years old, male).

The negative effects of dependent use centered on the loss of relationships and overall social functioning.

I started to lose the people around me. You sit with the people out of obligation not because you want to, which makes you feel lonely (Participant 10, 27 years old, male).

You keep staring at the blank wall for hours. You lose track of time. You can sleep for hours and even days without waking up (Participant 7, 35 years old, male).

The majority of participants reported withdrawal symptoms when attempting to self-detox and discontinue pregabalin use and described mental health difficulties in achieving cessation.

Every time I quit I develop a sort of social phobia, nightmares, and fear. Seriously, I can’t. I must take it (pregabalin) to get relief. Also, I had suicidal thoughts which made me want to take it again, and it caused me sexual dysfunction as well (Participant 1, 22 years old, male).

Others described a myriad of physical and mental conditions such as exhaustion, headaches, anxiety, depression, joint and muscle pains, shivering, lethargy, and avoidance of social interaction when attempting to reduce and cease use. These withdrawal symptoms contributed to users administering pregabalin again.

I felt shivering, headache, body ache, and heavy knees, but mostly it is the shivering that bothered me. All these symptoms made me want to take it (pregabalin) again (Participant 7, 35 years old, male).

I literally avoided talking to people even on the phone and I became inactive. I also suffered from headache, depression, shaky hands, and muscle ache, especially here (pointing to his knee). My mother told me that I sweated a lot during sleep and my temperature got high as well. After three days of quitting Lyrica®, I suffered from insomnia (Participant 11, 19 years old, male).

Two participants reported that when experiencing pregabalin withdrawal symptoms, they self-medicated with other drugs to alleviate symptoms.

I felt exhausted, moody, and avoided dealing with people. To alleviate these symptoms, I used to take Lexotanil®; it helped me to get rid of symptoms (Participant 5, 22 years old, male).

### Sourcing of Pregabalin

Four pregabalin users reported to have obtained their first supply from friends and then continued to source via purchasing from the community pharmacies while the other six sourced only from community pharmacies. One participant (no. 8) reported that his source of supply was through a prescription written by a doctor in the United Arab Emirates with physical purchase in a Jordanian community pharmacy. No participants reported street sourcing.

He (his friend) told me this is from the pharmacy without a prescription. Any pharmacy can sell it to you, and I went to the pharmacy he mentioned and bought it (pregabalin) (Participant 7, 35 years old, male).

The majority of participants were not aware of a street name for pregabalin products and usually requested it by its brand name like Lyrica® or Galica®. In contrast, three participants described the street name of Lyrica® or Epigab® as *Lulu*, *Laleze*, and the *trip* and were using these names among themselves.

We used to call Epigab® the “trip”, because as they say it takes you in a trip to another world (Participant 5, 22 years old, male).

The majority of participants described easy access and availability of pregabalin products in community pharmacies. Six participants got pregabalin by requesting a specific brand name or by the pharmacist himself/herself offering to give an alternative substitute. A minority of participants described some community pharmacies as refusing to sell the product or advising him that the drug was listed and required a prescription. Some pharmacists advised on alternative drugs.

Not all pharmacists would sell it (pregabalin). Some would refuse and explain it was listed and needed a prescription so I go and look for another pharmacy (Participant 11, 19 years old, male).

Many pharmacists sell it. I used to always buy Lyrica® from a specific pharmacy. Sure the pharmacist knows it’s for non-medical purposes because I used to buy it frequently and all he needed was money. Sometimes, he would say that the drug was out of stock, but he had other alternatives. That’s how I came to know about Epigab® and Regab® (Participant 2, 23 years old, male).

One participant described preferring to secure pregabalin in the morning when the owner or the responsible pharmacist was not available. In addition to that, he reported a difference in the price of the drug every time that he requested it from a pharmacy. Three of the participants described asking for a prescription from a physician.

In the UAE, they usually ask for a prescription but here [Jordan] no. I used to visit the doctor to obtain a prescription to buy it. I give him 100 dinars (500 dirhams) for the prescription. He is an Indian doctor, and he didn’t refuse to give me the prescription. But here [Jordan] it is normal, I can buy it from any pharmacy (Participant 8, 29 years old, male).I tried to secure a prescription from a doctor, but not from my side from another person’s side. My friend got me a prescription from a doctor that he knows. He is a private doctor (Participant 11, 19 years old, male).

## Discussion

The study provides us with a deeper overview about the problem of pregabalin abuse in Jordan from the perspectives of those affected by abuse and dependence. We recognize that the limitations of the study are its small-scale nature confined to two addiction centers in one Jordanian city and that reported experiences of pregabalin abuse may also have been clouded somewhat by the poly-substance repertoires of patients in these addiction treatment centers. The study, however, despite these restrictions reiterates the complexities of pregabalin abuse within the regulatory framework of availability in Jordanian community pharmacies. Of note was the demographic profile of the participants, similar to that reported elsewhere (Johnson et al. 2000; Jensen et al. 2016). The vast majority of participants were poly-drug abusers and had previous history of substance abuse (Schwan et al. 2010; Grosshans et al. 2013). It was encouraging to note the relative short-term use of pregabalin, despite negative experiences and difficulties in cessation. Routes of administration were similar to those reported in the literature; either taking tablets whole or cutting and snorting them is an example (Carrus and Schifano 2012; Millar et al. 2013).

Given the poly-pharming experience of the participants, the use of pregabalin in self-medication from synthetic cannabinoid effects, the use of pregabalin with other substances when seeking to potentiate the intoxicating effect, and the self-medication with other substances when attempting to self-detoxify from pregabalin are of concern. Synchronizing the inhibitory and anxiolytic effects of pregabalin by mixing pregabalin with sedatives is not a new behavior among pregabalin abusers as it was mentioned in the 2010s in different studies (Schwan et al. 2010; Schifano et al. 2011; Baird et al. 2014; Grosshans et al. 2013). This is also not surprising as it has been documented that pregabalin may be effective in treating withdrawal symptoms of drugs and alcohol associated with physical dependency (Freynhagen et al. 2016). The potential risk of pregabalin abuse or misuse in patients with a history of substance abuse should also be considered (Schjerning et al. 2016). Poly-substance abuse patterns are vital in contributing to the abuse liability of pregabalin (Schwan et al. 2010; Grosshans et al. 2013). Combinations are similar to those reported in other countries such as Austria and Greece in which pregabalin was frequently co-administered with alcohol, benzodiazepines, or cannabinoids (Papazisis et al. 2013; Yazdi et al. 2015). Combining pregabalin with sedative drugs is dangerous and unpredictable (Yazdi et al. 2015) since the user may feel more sedated and relaxed when taking them. Equally, consuming two psycho-depressant drugs can contribute to exaggerated heightened effects and may lead to overdose and death (Carrus and Schifano 2012).

Of particular concern also are the combination with new designer drugs such as synthetic cannabinoids, which carry particular risks and unpleasant withdrawal experiences for users (Palamar and Barratt 2016; Van Hout and Hearne 2017), and the use with Captagon (Van Hout and Wells 2016). This is a unique result found compared with previous literature. The only substances mentioned previously in the literature to be mixed with pregabalin were natural hashish, opioid, benzodiazepine, and alcohol, and this was attributed to the claim that pregabalin potentiates the effects of these drugs, which may have a similar effect of pregabalin (Carrus and Schifano 2012; Baird et al. 2014). The mixing of pregabalin with opioids by illicit drug users to enhance or prolong the effects of opioids has also been reported (Baird et al. 2014; Loftus and Wright 2014). Our study also commented on the enhanced use of cigarettes during pregabalin-taking episodes, a synergistic effect replicated by Driot et al. (2016).

Users also reported boosting the effect of pregabalin by combining it with various sweet drinks. The literature suggests that the sweetness activates the endogenous opioid system in animals and humans by inducing a release of β-endorphin and by increasing the binding affinity for opioids (Dum et al. 1983; Getto et al. 1984). Preference for stronger sweet solutions may represent a marker of alterations in brain systems that mediate rewarding responses to a variety of hedonic stimuli, including sucrose and alcohol (Kampov-Polevoy et al. 1997).

Participants reported a lack of awareness of adverse health consequences. Although there are several treatment centers in Jordan, the awareness regarding in-depth knowledge of substance abuse is lacking among high school students in Jordan, where young people constitute the majority of the Jordanian population (Haddad et al. 2010). Nonmedical use of a prescription drug may also be seen as being more socially acceptable than the use of illicit drugs such as cocaine or heroin (Hernandez and Nelson 2010). This coupled with the perception of legality and relative ease in securing pregabalin products or alternatives in community pharmacies underscores the need for heightened pharmacovigilance and educational efforts in community pharmacies (Jaber et al. 2015). Different factors (e.g., lack of pharmacist awareness about pregabalin abuse and prospect for economic gain to income) complicate the role of pharmacists in Jordanian communities. The lack of detection in urine screening additionally facilitates abusers to continue on pregabalin. The lack of detection might be due to the variability of urinary detection times of abused drugs, and differentiating new drug use from residual drug excretion could be difficult, especially after repeated or chronic drug usage (Spigset and Westin 2013).

## Conclusion

This qualitative study has illustrated that the problem of pregabalin abuse and misuse in Amman, Jordan, exists with many challenges and several complicating factors. Findings call attention to implementing effective community pharmacy-based interventions to raise patient and pharmacist awareness, address diversion, and ultimately reduce the abuse of pregabalin products.
